# Hospital and Institutional News

**Published:** 1918-08-10

**Authors:** 


### HOSPITAL AND INSTITUTIONAL NEWS.

### A DOCTOR TWICE A PRISONER.

For the second time, it is reported, Colonel H. B. Kelly, D.S.O., Royal Army Medical Corps, is a prisoner in Germany. In October 1914, when a captain, he was unofficially reported to be a prisoner, and was repatriated three summers ago. On June 15 last he was reported missing, and in a subsequent casualty list is included among the men who have been taken prisoner by the enemy.

### MORE MEDICAL M.P.s?

After an animated discussion the British Medical Association lately passed a resolution in favour of rendering financial support to assist the Parliamentary candidature of such medical men as may be approved. The fund will be voluntary, and it does not appear that the Association wishes to run its own candidates, but rather to start a voluntary fund out of which certain medical men who happen to stand may be helped. Probably the individual doctors who happen to be elected will benefit more than the Association. It was argued on the occasion of the discussion that Parliament was as bad an advertisement for doctors as it is good for lawyers. Certainly lawyers seem to enter Parliament for political rather than professional ambitions. Hitherto medical members have been few and far between, but when a Ministry of Health shall have become established, and an enlarged public medical service shall have followed in its train, then a political career will have a closer relation to professional prospects than it has had for doctors in the past. By that time, too, the British Medical Association will have a direct interest in medical members who can express its views in the matter of "health legislation" which will follow.

### FURTHER PRESENTATION TO A SECRETARY.

The retiring secretary of Devonshire Hospital, Buxton, who, as our readers will recall, has succeeded Dr. Basil Rhodes at the North Staffordshire Infirmary, has received a further presentation from the medical staff. This was an English gold lever watch inscribed as follows: "Presented to William Stevenson, Esq., as a mark of personal appreciation, by the medical staff of the Devonshire Hospital, Buxton. 19 July 1918."

### INFLUENZA AND HOSPITAL VISITORS.

In consequence of the epidemic of influenza, the board of management of Swansea Hospital has decided to close the institution to visitors. The hospital has also been virtually closed of late to patients in consequence of the absence of nurses, which for a fortnight prevented the admission of any save emergency cases. Meanwhile the salaries of the nursing staff are being raised. The sisters, at all events, are to receive an increase, and the salary of the *x*-ray sister has already been raised from £50 to £69.

### CO-ORDINATION OF MEDICAL SERVICE IN LEICESTER.

The Mayor of Leicester, Alderman Jonathan North, lately presided over a conference of representatives of the medical profession and of the health organisations of Leicester, to discuss the desirability of appointing an advisory committee to consider matters connected with the health of the town. The chairman of the board of management of the Leicester Public Medical Service presented a statement of the views of his board with regard to co-ordination in the matter of public health. In his opinion the value of the work now being performed by lay bodies would be greatly increased by an active and co-ordinated association with the medical profession. The Leicester Public Medical Service had been much appreciated, and in his judgment the facilities offered should be more largely made use of by local authorities. With this object in view, his board were contemplating many extensions and improvements of service—for instance departments for *x*-ray, electro-therapeutics, pathology, skin diseases, diseases of women and children, nervous diseases, and a consulting service. The Public Medical Service did not ask for financial aid, but encouragement, and the best way of bringing to fruition the ideals aimed at would be by the appointment of a committee representative of the various local interests, which should consider in a purely advisory capacity matters relating to the health of the town. A committee representing about thirty local public bodies and health organisations to be elected by those bodies was then agreed to. The new committee should help to avoid overlapping in the medical services of the town.

### THE UNPOPULARITY OF FACTORY SANATORIA.

The establishment of a factory sanatorium wherein employment could be found for tuberculous boot and shoe operatives under modified sanatorium conditions has again been suggested. This proposal was submitted by the Local Government Board to the Leicestershire and Northamptonshire County Councils and also to the Leicester and Northampton Town Councils. These authorities conferred with representatives of masters and men, and the conclusion is that a factory sanatorium is not practicable. The representatives desire that the Government should give financial support to workshops connected with existing sanatoria. The real objection seems to lie in a dislike of class institutions. Deep in human nature lies a passion for inequality, and the boast "I am as good as you" always means that "someone else is not as good as I."

### U.S.A. ARMY SCHOOL OF NURSING.

The committee on nursing of the U.S.A. General Medical Board, states the "Medical Record" of New York, has given its hearty support to the project of the Army School of Nursing, and the facilities at the command of the committee will be used in a recruiting campaign to secure suitable candidates for the school. The plan of the Army School of Nursing will be based on the methods that have been found to insure the best care of the sick in civil life. The standard will be that of high-class nursing schools such as John Hopkins, the Peter Bent Brigham, the Lakeside of Cleveland, Presbyterian, St. Luke's, etc. The plan provides that the headquarters of the school will be in the Surgeon-General's office, and the course of training will be given in the various base hospitals assigned as training camps, each of which is to be a complete unit. The plan provides that the course leading to a diploma is to extend over three years. The experience in the military hospitals is to provide surgical nursing, including orthopædic, eye, ear, nose, and throat, and medical nursing. Experience in children's diseases, gynecology, obstetrics, and public health nursing is given through affiliations with civil hospitals and visiting nursing organisations.

### FIRST AID BY AEROPLANE.

The Americans are understood to be organising a system of first aid by aeroplane. The doctor takes the place of the observer. The immediate object seems to be to render prompt assistance to men whose aeroplanes have been seen to fall from the flying aid posts which are part of the system. According to *Pearson's Weekly* a fire-engine and an ambulance follow, so that the effects of accident are remedied by every means. An airman's Ambulance Service has an up-to-date sound which we hope will fulfil expectations.

### THE MECHANICAL HAND.

In the Edinburgh College of Art an interesting exhibition has been held of the more complicated mechanical devices which have been invented to supplement physical disabilities. These require to be seen and handled if their capacity is to be understood, a description, even one with illustrations, cannot convey a real idea of their ingenuity. The development which has taken place in the artificial hand is perhaps the most remarkable. Each country seems to have added some improvement, the most complicated of which is the invention of Dr. Puttis of Bologna, whereby the severed muscles are connected by artificial sinews with a mechanical hand. It is claimed that the hand then opens and shuts as the brain directs much as if it were a live one. Whether or not there is a natural limit to mechanical devices of this kind will be proved at the end of the war, when the need for them will have carried ingenuity, through the stiff test of experience, to its highest contemporary point.

### CATERING FOR SOLDIERS.

The Central V.A.D. Committee has issued a book of recipes and catering notes for use in auxiliary hospitals. Since catering is one thing and cooking another, the book is intended not only for cooks, but for commandants. No one who has been inside an auxiliary hospital but knows that the men have prejudices for and against the simplest dishes, and that there is an art in combining respect for their often monotonous preferences with the change and variety that must be attempted, so far as means allow, for the other sections of the staff. Any commandant or interested person should apply for a copy of the work to the V.A.D. Department, Devonshire House, Piccadilly.

### THE "SPRINGBOK MAGAZINE."

The amalgamation of the South African Military Hospital with the Richmond Military Hospital, Grove Road, is now complete, and the institution has become the centre of various auxiliary hospitals which are placed under its administration. In the July number of the *Springbok Magazine* the account of "Richmond Park and its Associations" is continued by Mr. E. A. Barkas, the borough librarian of Richmond, and the historical portraits which accompany it do something to bring the chequered history of the park before our eyes. An unusual form of recreation is provided by the Rambling Club, which, generally under the guidance of Mr. Barkas, visits such places as Sudbrook Park, Ham, and Petersham. The magazine is thickly interspersed with advertisements, and the list of patients is of general interest to all who have friends in the South African contingents.

### A CALL FOR ACTION BY THE PHARMACEUTICAL SOCIETY.

The action taken regarding dispensing of medicines by unqualified persons, as reported in The Hospital (page 340), by the chairman of the Public Pharmacists' Association, has been amply justified by two occurrences recently. In one case (The Hospital, page 352) the inspector of the Local Government Board sent a report to the Worcester Guardians, pointing out the fact that no qualified dispenser was engaged at the infirmary, the bulk of the medicines being "dispensed" by the superintendent nurse. It is true the statement was made "that this nurse acted under the direction of the medical officer," but, in these days of shortage of medical help, one would imagine that it would be preferable to engage a properly qualified dispenser. If the amount of dispensing does not warrant the employment of a full-time officer, arrangements might be made with a local pharmacist to do the work. Evidently the L.G.B. inspector is not quite satisfied with the present arrangements for dispensing at Worcester. The second case of dispensing by unqualified persons is much more serious, in that it has resulted in the death of a cadet of the R.A.F. At the inquest (as reported in the *Pharmaceutical Journal*) this cadet, a patient in the camp hospital, was given a dose of carbolic acid in mistake for quinine mixture. The evidence showed that a corporal acted as dispenser, and he gave carbolic acid to a V.A.D. worker in a "sauce-bottle" labelled "Quinine mixture," and this poison was administered with fatal results. The jury expressed the view that in future it was essential that the camp should have a qualified dispenser, and the Coroner, agreeing with the jury, is reported to have said: "There are properly qualified chemists in the Army pushing wheelbarrows, etc., about, whereas they might be usefully employed in their important work." Now that hospital pharmacists are being "called up," the Army authorities should, for the warriors' defence, utilise their technical training in the dispensing of medicines under Army control. The committee appointed by the Pharmaceutical Society, as suggested by Mr. A. H. Jenkin, should go thoroughly into the questions raised by the employment of unqualified persons in this connection in the interests of everybody. Do this at once, and save any further loss of life from this cause.

### IMPORTANT GIFT TO STOCKTON HOSPITAL.

To celebrate the sixtieth anniversary of his wedding, Sir Robert Ropner, Bart., M.P., has given £10,000 to the Stockton and Thornaby Hospital. The money is intended to form the nucleus of a rebuilding or extension fund, and, the donor says, "to mark the many happy years I and Lady Ropner have spent in the district." It is possible that a new hospital will be built after the war, but Sir Frank Brown, the chairman, and the committee are not fettered by precise conditions attached to the gift.

### A MEDICAL MISSION IN DIFFICULTIES.

The public hears too little of the interesting and often remote work of the medical missions, and in difficult times like the present such missions are apt to suffer from want of support. For instance, the Zenana Bible and Medical Mission is groaning under a heavy debt. Yet the announcement of this debt will be to most people the first piece of news which the mission has allowed to reach them. It should not be so. A medical mission in India, particularly a Zenana Medical Mission, must have a fund of interesting details of life and work in India, the tale of which, had it ever been made public, would have secured continuous interest and support. We should almost suppose that medical missions had something to conceal, from the few and grudging statements of their doings which occasionally are allowed to filter through the veil of obscurity in which they hide their work. All this is want of intelligence, and when we learn of their financial troubles we feel bound to remind them how little they have ever done to interest anyone in their affairs. Cannot Lord Kinnaird, the treasurer, or Sir Andrew Wingate, the chairman, now that they have their backs to the wall, so to speak, remedy this state of affairs, and let the public know what work they do and how they do it? The life of a medical mission in India must be full of interest to those who are acquainted with the conditions of its work.

### WOMEN AND HEALTH APPOINTMENTS.

Leeds Corporation Health Committee has appointed Miss E. M. Smith, of the Royal College of St. Katherine, Poplar, as assistant to the Chief Woman Inspector at a salary of £130 a year, with war bonus and allowance for uniform. Miss Lilly Barnham has been appointed health visitor at a salary of £104 a year, with war bonus, etc.

### VOLUNTEER NURSES IN INFIRMARIES.

Bethnal Green Guardians are still in difficulties in regard to staffing the infirmary. The committee, which has already reported upon the engagement of help from nursing homes, now recommends the engagement of Miss Gilligan, of the Parmiter Nursing Home, Highgate, at a fee of 2½ guineas a week, and Miss Pearce, from Sister Truan's Nursing Home, Leytonstone, at a fee of 2 guineas a week. Miss Maud Carter, an assistant nurse from the Poor-Law schools, has taken up duty temporarily at the infirmary until the nursing staff is increased. The committee reports that nine ladies have offered to give their services in the wards as voluntary workers for several hours a week, and a vote of thanks has been accorded them for their services. Regarding Miss Saxby, the matron, the committee reports that she has left the London Fever Hospital and that it is expected that the other patients will be discharged shortly. It is suggested that eight weeks' leave of absence be granted to all the typhoid cases from the time they leave the hospital, and that the sum of 25s. a week each be paid to them in lieu of rations and other emoluments which they would have received had they returned direct to duty.

### THE APPOINTMENT OF WARD ASSISTANTS.

In reference to additional assistance in the infirmary wards, the committee, on the report of the medical superintendent, recommends that three women be appointed for a period of three months, to be known as ward assistants, their duties being to lay out and prepare for burial bodies of patients dying in the infirmary, and when not engaged in this work to be employed in washing, bathing and feeding patients, or mending clothes, such persons to be non-resident and paid £1 13s. 6d. per week, inclusive of war bonus.

### VACCINATION OFFICERS' FEES OR SALARY.

The Local Government Board have sanctioned the arrangement of the Southwark Board of Guardians to pay their vaccination officer a fixed salary of £160 a year instead of him receiving payment by fees under Article 20 of the Vaccination Order, 1898. The facts were reported in The Hospital on July 13, page 319. The arrangement is a just and equitable one, considering that the appointment is practically a full-time one, and that owing to the reduction in the birth rate, together with the increase in the number of persons who have of late years raised a conscientious objection to vaccination, the fees have gradually fallen off. There is little doubt that other Metropolitan Boards of Guardians will follow the example of Southwark and Lambeth. In the latter borough the payment of fixed salaries to the vaccination officers has been in force some years.

### GRAVESEND HOSPITAL FETE.

JULY 31 and August 1 were great days for the Gravesend Hospital, when the open-air fête, in the preparation of which the friends of the hospital have been busily engaged for some time, was opened on the first day by H.R.H. Princess Arthur of Connaught, and on the second day by the Countess of Darnley. Fortunately, the weather was a little better than most districts have been experiencing lately, and a large number of people attended the function and enjoyed the full programme of events, including the music provided by the bands of the New Zealand Medical Corps and the Royal Marines. Mr. and Mrs. R. H. Foà, who had in other ways done much to secure success, made it sure by handing over a special collection of £1,192 gathered from their friends towards the funds of the hospital. One of the stalls at which one could buy assorted articles secured special attention by the topical notice that " No coupons " were required to secure the goods, and at another visitors were allowed, on payment of 1s., to sign their names on a Union Jack destined to be presented to the hospital; the Princess enhanced the value of this souvenir by adding her name early in the proceedings. So far as can be ascertained, the total proceeds, up to the present, amount to £2,317, and the hospital is to be cordially congratulated on the success of a splendidly organised money gatherer.

### PRESENTATION TO REV. A. LOMBARDINI.

ON leaving Kensington Infirmary to take up work as a Chaplain to the Forces, the Rev. A. Lombardini was the recipient of many presents. From the past and present members of the Kensington Infirmary Nurses' League Mr. Lombardini received a handsomely fitted dressing case, and with the pennies collected by the old men and women a fountain pen and field water-bottle were purchased. The members of the choir and several others interested in the institution also took advantage of the opportunity to show their appreciation for the work carried out by the Chaplain during his five and a-half years at the church of St. Elizabeth. A small party was organised by Miss Alsop on Friday afternoon, and many of the Kensington Guardians were present. In the evening a concert arranged by the staff was held, at the conclusion of which speeches were made by Dr. Remington Hobbs, the matron, and senior nurse. Mr. Lombardini leaves with the sincerest good wishes of everyone, and a warm welcome on his return is assured to him. In the absence of the editor, the *Kensington Nurses' League Journal* will of necessity have to be of a " war-time " nature.

### MISAPPREHENSION OF SOUTHWARK GUARDIANS

THE Southwark Board of Guardians are reported to claim to be in an undignified position. They seem to have forgotten the arrangements for the use of Poor-Law institutions for military patients. The Guardians can always have advances from the Command paymaster without reference to the commandant (except as regards the initial advance), and it is unnecessary for them to obtain the latter's sanction for payments on account after the first time. The passing of expenditure actually incurred is, of course, on a different footing. The Guardians' accounts are examined by the Local Government Board itself in the first instance, after they have been submitted to the commandant, who will call attention to any items which he considers questionable. When the Guardians desired a payment on account with respect to expenditure at their infirmary (now a military hospital), after the first payment on account, the arrangement is not that they should obtain a certificate from the officer in command that their request is a reasonable one. This supposed indignity which the Southwark Board of Guardians have resented by appealing to the War Office to release them from what has become an undignified position appears therefore to relate to something which has no existence in fact under the arrangements made. No doubt the confusion which seems to have occurred in the minds of the Guardians between the procedure on claiming an advance and on presenting their accounts for audit has led to misunderstanding, which should never have occurred on the part of a body which conducts all its business on a businesslike footing. It is satisfactory to learn on authority that no such position as the Southwark Guardians have denominated as undignified can be created, providing the Board take steps to free their minds from confusion and keep their arrangements for the use of Poor-Law institutions constantly in mind. The Guardians may rest assured that they will always find smooth working and business arrangements in all matters for which the Financial Secretary to the War Office, the Rt. Hon. H. W. Forster, M.P., may be in any way responsible.

### THIS WEEK'S DRUG MARKET.

THE market has been practically at a standstill since the latter part of last week, but an early renewal of activity is probable in view of the fact that at the present time buyers do not buy far ahead of their immediate requirements. A small business has been done in Curaçoa aloes at firm rates. Siam gum benzoin is dearer, and the value of Sumatra gum benzoin is well maintained. A fair business has been done in honey at irregular prices. The value of sarsaparilla is not quite so firmly maintained. Witch hazel leaves have been sold at extraordinarily high prices. There is no change in the position of bromides, but it is not improbable that sodium and ammonium bromides will recede in price, as the demand for these two salts will be relieved in consequence of the more plentiful supplies of potassium bromide and its reduced price. The value of quinine is firmly maintained. Artificial oil of wintergreen continues to tend downwards in price. Sal ammoniac is dearer.

### TO OUR READERS.

CONTRIBUTIONS are specially invited from any of our readers to these columns. They should deal with topical subjects and news. They must be authenticated for the information of the Editor only. The minimum payment if published is 5s. There is no hard-and-fast rule as to space, but notes of about twenty lines in length are preferred.

